# Expotype–phenotype resilience and multimodal aging clocks

**DOI:** 10.1002/ctm2.70558

**Published:** 2025-12-26

**Authors:** Hernan Hernandez, Agustin Ibanez

**Affiliations:** ^1^ Latin American Brain Health Institute (BrainLat), Universidad Adolfo Ibañez Santiago de Chile Chile; ^2^ Cognitive Neuroscience Center, Universidad de San Andrés Buenos Aires Argentina; ^3^ Department of Biophysics Istanbul Medipol University Istanbul Turkey; ^4^ Global Brain Health Institute (GBHI) Trinity College Dublin Dublin Ireland

## 1 MULTIMODAL RESILIENCE IN AGING

Aging trajectories vary widely across individuals, even under comparable biological and environmental pressures, yet most biomedical frameworks prioritize vulnerability over protection. This perspective proposes a shift towards identifying resilient expotype–phenotypes, defined by combinations of exposures and individual adaptive responses that support unexpectedly healthy aging. We propose multimodal aging clocks (focusing on delayed agers) to address resilience and its phenotypic and expotype contributions. Building on recent evidence from global exposome analyses^1^, ^2^, ^3^, ^4^, multimodal aging clocks^1^, ^5^, ^6^, ^7^, ^8^ and neuroecological frameworks^2^, ^9^, we argue that resilience offers an essential dimension for precision brain health.

The exposome^1^, ^10^ captures the totality of physical, social and sociopolitical exposures across the lifespan, exerting marked influences on biological, systemic and cognitive health. This multidimensional construct provides a foundation for defining expotypes^11^, the characteristic combinations of exposures that shape individual risk or protection^12^. Aging clocks (epigenetic clocks, proteomic or multi‐omic clocks, brain clocks and biobehavioural clocks) quantify biological aging relative to chronological time, enabling direct assessment of how exposures modulate aging trajectories. These tools reveal that diverse exposures, from pollution and temperature peaks to structural inequalities and political instability, accelerate biological aging,^1^, ^2^, ^6^ whereas enriching environments, cognitive stimulation and social cohesion may delay it. Together, they provide a framework for evaluating how cumulative exposures influence aging across datasets, populations and biological systems^12^, ^13^. Thus, the exposome and aging clocks may jointly enable a more mechanistic assessments of how protective and adverse exposures shape biological aging across systems.

Our recent *Nature Medicine* study illustrates how biobehavioural age gaps (BBAGs) – the discrepancy between predicted age from protective/risk factors and chronological age – capture delayed or accelerated aging across 40 countries^1^. BBAGs were estimated using a Gradient Boosting Regressor with 10‐fold cross‐validation to predict age from biobehavioural factors (risk and protective) in >160 000 participants. The age gap was computed as predicted minus chronological age, with negative values indicating delayed and positive values indicating accelerated aging. To correct regression‐to‐the‐mean, gaps were residualized against chronological age using coefficients from the training set and applied to the test set. Delayed BBAGs were linked to favourable exposomes: cleaner air, inclusive migration contexts, structural and gender equality, and democratic stability. These findings illustrate a neurosyndemic, neuroecological processes, where environmental, behavioural and political stressors converge to shape vulnerability or resilience^2^, ^13^. They also demonstrate the feasibility of connecting macrostructural features, such as governance, income distribution and collective stress, to individual‐level metrics of aging^13^, ^14^, ^15^. Integrating exposome dimensions with individual clocks therefore allows the tracing of multiple pathways through which global conditions embed into biological trajectories^16^.

## 2 CHANGING THE FOCUS FROM VULNERABILITY TO RESILIENCE WITH MULTI‐AGING CLOCKS

Most exposome and aging‐clock studies focus on accelerated aging, disease risk and vulnerability. Yet across populations (particularly those enduring adversity), some individuals exhibit unexpectedly delayed aging or preserved function. Such phenotypes cannot be explained by individual risk factors alone; they point to resilience mechanisms capable of counteracting adverse exposomal conditions. These individuals reveal protective expotypes: interactions of exposures, behaviours and biological processes that sustain healthy trajectories beyond what risk‐based models predict. Understanding these high‐resilient individuals is critical for precision medicine. They provide evidence about protective mechanisms, context‐specific adaptations, individual heterogeneity and compensatory biological dynamics that remain invisible when scientific inquiry centres solely on vulnerability.

Resilience is inherently multimodal, involving biological, psychological, cognitive, social and environmental domains. Recent work^17^ shows that genetic factors, epigenetic regulation, inflammation and metabolic flexibility interact with cognitive reserve, emotional regulation, coping strategies, community bonds and environmental factors to influence brain aging. Physical and social environments, including nutritional stability, pollution, green spaces, governance quality and neighbourhood cohesion, may further modulate these interactions. For example, favourable omic signatures, such as reduced inflammatory burden, may buffer against pollution‐related risks; psychological resilience may mitigate socioeconomic stress; and strong social networks may support metabolic and cognitive stability in environments of chronic adversity. Resilience is not a single trait but a synergetic architecture^17^, integrating multiple processes that collectively preserve healthy aging in challenging contexts.

Multimodal aging clocks offer a powerful means to quantify resilience by identifying delayed aging relative to expected trajectories. Epigenetic clocks capture reduced biological wear; BBAGs signal favourable biobehavioural patterns; and brain clocks reveal structural or functional maintenance. Individuals with extreme delayed clocks represent *maximal resilience phenotypes*, while those showing moderate delays despite multiple risk exposures reveal compensatory protection. Modelling these patterns within synergetic^12^ and syndemic^13^ frameworks can disentangle how protective factors interact, rather than treating resilience as a unidimensional construct. Moreover, digital twins^8^ and biophysical computational models^12^ can generate synthetic scenarios to test how combinations of protective exposures (diet, exercise, education, community engagement, environmental quality) interact with omic and brain dynamics. These simulations can support individualized predictions and scalable interventions that address both person‐level and context‐level variability. In brief, delayed multimodal aging clocks can quantify resilience, enabling mechanistic and individualized analysis of how protective factors buffer aging.

Reframing the exposome from a burden‐centred to a resilience‐centred concept offers multiple benefits. Firstly, it may help to explain why some individuals sustain exceptional health despite adversity and may allow identifying therapeutic pathways underlying these protective adaptations. Secondly, genetic^18^, biological^19^, lifestyle^5^, ^9^ and psychological^20^ protection may reveal resilience as a pluricausal phenomenon rather than a single‐level trait.^17^ Thirdly, high‐resilient individuals help refine precision brain health models: their trajectories provide boundary cases that improve predictions, optimize risk stratification and support individualized interventions across heterogeneous settings. This shift may expand aging research beyond deficit accumulation, emphasizing adaptive processes that may be leveraged to design interventions targeting protection rather than merely reducing risk.

## 3 CONCLUSIONS: A NEW RESEARCH AGENDA

**FIGURE 1:**
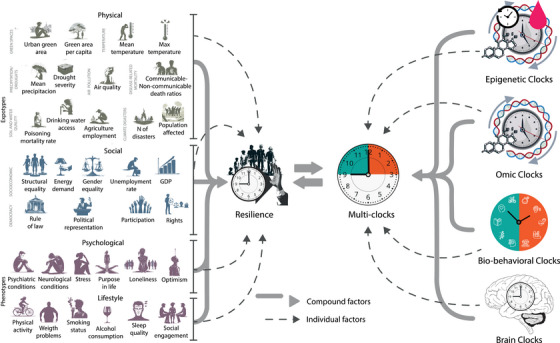
Expotype–phenotype contributions to resilience via multimodal aging clocks. The figure illustrates how two major domains of the expotype (social and physical exposome) and two phenotypes (psychological trails and lifestyle factors) may co‐contribute to resilience processes that shape delayed biological aging. Each domain encompasses exposures: the expotype involves compound factors spanning green areas, air quality, temperature, drought severity, precipitation patterns, soil and water quality, climate‐related disasters, structural and gender equality, unemployment rate, GDP, rule of law, political representation, participation rights and drinking‐water access, while the phenotype includes individual traits such as mental or neurological conditions, demographics, as well as lifestyle factors such as physical activity, weight problems, smoking status, alcohol consumption, sleep quality and social engagement. Together, these configurate internal and external influences acting on an individual's resilience. Converging arrows highlight how these domains collectively modulate resilience, conceptualized as the capacity to maintain or regain function despite adversity. Resilience, in turn, can be measured by multimodal aging clocks, including epi/genetic/omic clocks, biobehavioural age gaps (BBAGs), and brain structural/functional clocks. These clocks quantify delayed or accelerated aging and serve as mechanistic markers of how protective or adverse expotypes shape aging trajectories.

A future research programme (Figure 1) is needed to systematically identify resilience underlaying complex expotypes and phenotypes: multivariate patterns of exposures, biological states, and behaviours that jointly support delayed aging^12^, ^13^. Moving beyond isolated predictors, this approach requires integrating multimodal clocks, exposome metrics and resilience indicators into synergetic models^12^, ^13^ capable of detecting how positive environments interact with individual conditions to shape advantageous trajectories. These approaches must also track how protective exposures compensate for specific vulnerabilities (i.e. metabolic risk, educational disadvantage, pollution or sociopolitical instability) and how these interactions differ across contexts. Such precision will allow interventions to move from universal prescriptions towards context‐sensitive combinations tailored to local conditions and individual profiles. Studying protective expotypes together with phenotypes will inform strategies that promote healthy aging by enhancing resilience through synergistic biological, psychological and environmental pathways.

## CONFLICT OF INTEREST STATEMENT

The authors declare no conflicts of interest.
